# *Bergenia pacumbis* from Nepal, an astonishing enzymes inhibitor

**DOI:** 10.1186/s12906-020-02989-2

**Published:** 2020-06-26

**Authors:** Bishnu Prasad Pandey, Suman Prakash Pradhan, Kapil Adhikari, Saroj Nepal

**Affiliations:** 1 Department of Chemical Science and Engineering, Kathmandu University, PO Box No. 6250, Dhulikhel, Kavre Nepal; 2 H-plant Private Limited, Sanepa, Lalitpur, Nepal

**Keywords:** *Bergenia pacumbis*, Antioxidant, Enzyme inhibition, Major metabolites, HRMS

## Abstract

**Background:**

The *Bergenia* species are perennial herbs native to central Asia, and one of the most promising medicinal plants of the family Saxifragaceae which are popularly known as ‘Pashanbheda’. The aim of this study was to evaluate antioxidant and *α-*amylase, *α-*glucosidase, lipase, tyrosinase, elastase, and cholinesterases inhibition potential of *Bergenia pacumbis* of Nepali origin collected from the Karnali region of Nepal.

**Methods:**

The sequential crude extracts were made in hexane, ethyl acetate, methanol, and water. Antioxidant activities were analyzed by 2,2-diphenyl-1-picrylhydrazyl (DPPH) and 2,2′-azino-bis (3-ethylbenzothiazoline-6-sulfonic acid) (ABTS) assay. The *α-*amylase, *α-*glucosidase, lipase, tyrosinase, elastase, acetylcholinesterase, and butyrylcholinesterase inhibition were analyzed by the 3,5-Dinitrosalicylic acid (DNSA), p-Nitrophenyl-α-D-glucopyranoside (p-NPG), 4-nitrophenyl butyrate (p-NPB), l-3,4-dihydroxyphenylalanine (L-DOPA), N-Succinyl-Ala-Ala-p-nitroanilide (AAAPVN), acetylthiocholine, and butyrylcholine as a respective substrate. The major metabolites were identified by high performance liquid chromatography with electron spray ionization- quadrupole time-of-flight mass spectrometry (HPLC-ESI-QTOF-MS) profiling.

**Results:**

Our results revealed the great antioxidant ability of crude extract of *B. pacumbis* in ethyl acetate extract against both DPPH (IC_50_ = 30.14 ± 0.14 μg/mL) and ABTS (IC_50_ = 17.38 ± 1.12 μg/mL). However, the crude methanol extract of *B. pacumbis* showed the comparable enzymes inhibitions with standard drugs; *α-*amylase (IC_50_ = 14.03 ± 0.04 μg/mL), *α-*glucosidase (IC_50_ = 0.29 ± 0.00 μg/mL), lipase (IC_50_ = 67.26 ± 0.17 μg/mL), tyrosinase (IC_50_ = 58.25 ± 1.63 μg/mL), elastase (IC_50_ = 74.00 ± 3.03 μg/mL), acetylcholinesterase (IC_50_ = 31.52 ± 0.58 μg/mL), and butyrylcholinesterase (IC_50_ = 11.69 ± 0.14 μg/mL). On the basis of HPLC-ESI-QTOF-MS profiling of metabolites, we identified major compounds such as Bergenin, Catechin, Arbutin, Gallic acid, Protocatechuic acid, Syringic acid, Hyperoside, Afzelechin, Methyl gallate, Paashaanolactone, Astilbin, Quercetin, Kaempferol-7-O-glucoside, Diosmetin, Phloretin, and Morin in methanol extract which has reported beneficial bioactivities.

**Conclusion:**

Our study provides a plethora of scientific evidence that the crude extracts of *B. pacumbis* from Nepalese origin in different extracting solvents have shown significant potential on inhibiting free radicals as well as enzymes involved in digestion, skin related problems, and neurological disorders compared with the commercially available drugs.

## Background

The *Bergenia* species are perennial herbs native to central Asia, and one of the most promising medicinal plants of the family Saxifragaceae which are commonly known as ‘Pashanbheda’. Because of anti-lithiatic and diuretic activities, different species of *Bergenia* are used to treat kidney and urinary bladder stones and root powder is used to cure diarrhea, dysentery, thirst, vomiting, and indigestion in traditional medicine practice in Nepal, India, and China [1, 2]. Numerous pharmacological activities such as antipyretic, antioxidant, antilithiatic, antiplasmodial, antitussive, antiulcer, antidiabetic, hepatoprotective, hemorrhoidal, analgesic, insecticidal, anti-inflammatory, antimicrobial, and diuretic properties have been reported in different species of *Bergenia* [3–6]. Although a variety of secondary metabolites have been identified from different parts of *Bergenia* species [6, 7], the major bioactive phenolics compounds mainly concentrated in their roots; bergenin, arbutin, and gallic acid, are principal contributor of the therapeutic properties of *Bergenia* species [8–10] that leads to variation in their medicinal activities.

Diabetes mellitus (DM) is a life-threatening metabolic disorder characterized by chronic hyperglycemia resulting from defects in insulin secretion, insulin action, or both [11, 12]. *Alpha*-glucosidase and *alpha*-amylase are the important enzymes involved in the digestion of carbohydrates; *α*-amylase is involved in the breakdown of long-chain carbohydrates and *α*-glucosidase breaks down starch and disaccharides to glucose and helps in intestinal absorption [13, 14]. The *α-*amylase and *α-*glucosidase inhibitors help to reduce the rate of digestion of carbohydrates and thus type 2 diabetes mellitus (T2DM) complications [15]. On the other hand, an uneven accumulation of fat through the disproportion intake of calories and their utilization results in obesity which is becoming a major public health concern [16]. In the present context, the complex interactions of genetic, behavior, and surrounding environment with economic and social status and lifestyles of human beings are responsible for the up surging of T2DM complications [17, 18]. Moreover, obesity enables the development of different metabolic disorders such as diabetes, hypertension, and cardiovascular diseases [19–21]. Suppressing the absorption of dietary lipids in the gastrointestinal tract is one of the best option to overcome the obesity problems which can be accomplished by inhibiting pancreatic lipase enzyme that is responsible for the digestion of fats consumed in the regular diets [22, 23].

Although, melanin can protect tissues from oxidative and chemical stress [24], the excessive production of melanin causes various skin related problems. Melanin biosynthesis occurs in the melanocytes through a series of enzymatic and chemical reactions. Tyrosinase is a copper-containing monooxygenase that is widely distributed in microorganisms, animals, and plants which contribute to the melanin synthesis pathways [25]. Tyrosinase is frequently used in cosmetic and food industries as an anti-browning agent [26]. However, a high amount of tyrosinase might leads to hyper melanogenesis. Elastase is a primary enzyme responsible for the breakdown of elastin, which has unique elastic recoil properties and vital for giving elasticity to the skin [27–29] and other extracellular matrices (ECM) proteins which are responsible for providing support, segregating tissues from one another, cell migration, gene expression, and regulating intercellular communication [29, 30]. Finding inhibitors of tyrosinase and elastase enzymes can be useful to prevent skin pigmentation and loss of skin elasticity which works as skin whitening and anti-aging agents correspondingly.

Alzheimer’s disease (AD) is characterized by the loss of cholinergic neurons that alters the brain activity and cause cognitive impairment which is mainly caused by the progressive reduction of acetylcholine. Thus, inhibition of acetylcholinesterase and butyrylcholinesterase has been considered as a potential target in the treatment of AD [31]. To the best of our knowledge, no such investigation of the biochemical activity of *B. pacumbis* has been carried out to date. In this study, we focused on the profiling of major metabolite constituents and reported the in-vitro enzymes inhibition activity of *Bergenia pacumbis* (Buch.-Ham. ex D. Don) C.Y. Wu & J.T. Pan of Nepalese origin collected from the Karnali region of Nepal in the different solvent extracts for the first time which are beneficial in gaining scientific evidence in curing the negative health impacts of daily life-threatening enzymes; *α-*amylase, *α-*glucosidase, and lipase that involved in digestion, tyrosinase and elastase that involved in skin related problems, and acetylcholinesterase and butyrylcholinesterase that involved in neurological disorders.

## Methods

### Chemicals used

Different chemicals reagents used in this study were purchased from different chemical suppliers. Quercetin, 2, 2-Diphenyl-1-picrylhydrazyl (DPPH) and 2, 2′-Azino-bis (3-ethylbenzothiazoline-6-sulfonic acid) diammonium salt (ABTS), *α-*amylase from procine pancreas (Type VI-B), Tyrosinase from mushroom, Lipase from procine pancreas (Type II), 4-nitrophenyl butyrate (p-NPB), Orlistat, Acarbose, *α-*glucosidase from *Saccharomyces cerevisiae* (Type-I), p-Nitrophenyl-α-D-glucopyranoside (p-NPG), Sodium carbonate, Kojic acid, l-3,4-dihydroxyphenylalanine (L-DOPA), N-Succinyl-Ala-Ala-p-nitroanilide (AAAPVN), Porcine pancreatic elastase (PPE), Galantamine, Acetylcholinesterase (AChE), Butyrylcholinesterase (BChE), Acetylcholine iodide, Butyrylcholine iodide, 5,5′-dithiobis-(2-nitrobenzoic acid) (DTNB), Acetonitrile (LC-MS grade), Methanol (LC-MS grade), Formic acid (analytical grade), and Acarbose from Sigma-aldrich (USA). The 3,5-Dinitrosalicylic acid (DNSA) from HIMEDIA (India) and Sodium-potassium tartrate from Merc (Germany). Methanol, Sodium Hydroxide, Dimethyl sulfoxide (DMSO), and Starch from Fisher Scientific (India). Aluminum Chloride, Hexane and Gallic Acid from LOBA Chemie (India).

### Plant collection and authorization

The plant and its rhizomes were collected from Rimi village of Chankheli Rural municipality, Humla District, Karnali Province of Nepal (29°07′52“N and 82°30’50”E) in August 2018 and identified in Nepal National Herbarium and Plant Laboratory (KATH) Godhawori, Lalitpur, Nepal with reference letter number of 2074/2075–230.

### Sample preparation

The collected plant sample was dried at room temperature and made powdered by grinding in an electrical grinder. The crude extract of *B. pacumbis* was extracted in hexane-ethyl acetate-methanol-water system where extracts dependent on the polarity of the solvent, the chemical nature of the extracted compound, the plant matrix, and presence of interfering substances. Ten-gram powder sample was macerated in 100 mL of hexane and kept overnight on a shaker at 150 rpm at room temperature. On the next day, the entire mixture was filtrated and collected. The residue was further sequentially extracted with ethyl acetate, methanol, and water successively. The filtrated of each fraction were concentrated to dryness by evaporating on vacuum evaporator (hanil Modul 4080C). The extracted crude samples were kept in an airtight glass vial and stored at 4 °C until use.

### Antioxidant activities

#### DPPH assay

In-vitro antioxidant potential of the different extracts of *B. pacumbis* was assessed by 1,1-diphenyl 1–2-picryl-hydrazyl (DPPH) assay accordingly with Jha et al. [32]. One milliliter of plant extract of different concentrations was added in 3 mL of the DPPH solution (100 mM) and incubated for 30 min in the dark. Absorbance was measured at the wavelength (λ) = 517 nm in a UV-visible spectrophotometer (UV-1800, SHIMADZU). Gallic acid and methanolic DPPH were used as reference and positive control respectively. The following formula was used to calculate percentage of radical scavenging (%RSA).

$$ \%\mathrm{RSA}=\frac{\  Absorbance\ of\ Control- Absorbance\ of\ Test}{Absorbance\ of\ Control}\ast 100\% $$

#### ABTS assay

In-vitro 2, 2′-azino-bis (3-ethylbenzothiazoline-6-sulphonic acid) (ABTS) scavenging assay was performed as by Liyanaarachchi et al. [33]. An aliquot of 1 mL of plant extract of varying concentrations was mixed with 3 mL of ABTS working solution and incubated for 10 min in the dark and the decrease in absorbance was recorded at λ = 720 nm in a UV-visible spectrophotometer. Gallic acid and 50% methanol were used as reference and positive control respectively. The percentage of ABTS scavenging was determined by the following formula.

$$ \%\mathrm{RSA}=\frac{Absorbance\ of\ Control- Absorbance\ of\ Test}{Absorbance\ of\ Control}\ast 100\% $$

### Alpha-amylase inhibition activity

The DNSA method was used to determine *α-*amylase inhibition activity with a slight modified procedure of Wickramaratne et al. [34]. Briefly, 200 μL plant extract of varying concentrations and 200 μL 3 unit/mL α- amylase were mixed and incubated for 15 min at 37 °C. Two hundred microliter of 1% starch solution was added on it and further incubated for the next 5 min at 37 °C, then 200 μL of DNSA solution was added on it and heated for 10 min at 90–95 °C in order to terminate the reaction and the final volume was adjusted to 5 mL by the addition of distilled water. The absorbance was measured at λ = 540 nm in a UV-visible spectrophotometer. 1% DMSO and Acarbose were used as positive control and reference respectively. The following formula was used to calculate the percentage of *α-*amylase inhibition.

$$ \%\alpha -\mathrm{amylase}\ \mathrm{inhibition}=\frac{Absorbance\ of\ Control- Absorbance\ of\ Test}{Absorbance\ of\ Control}\ast 100\% $$

### Alpha-glucosidase inhibition activity

The *α-*glucosidase inhibitory activity was measured by using p-NPG as a substrate with a modified method from Elbashir et al. 2018 [12]. Briefly, 10 μL of the sample solution with varying concentrations were mixed with 60 μL of phosphate buffer (0.2 M pH 6.8) and 10 μL of *α-*glucosidase (1 U/mL) in phosphate buffer and incubated for 5 min 37 °C. 20 μL of p-NPG (4 mM) was added to the mixture and incubated further for 12 min and the absorbance was measured at λ = 405 nm in a UV-visible spectrophotometer. 1% DMSO and Acarbose were used as positive control and reference respectively. The percentage inhibition of *α-*glucosidase was calculated as follows.

$$ \%\alpha -\mathrm{glucosidase}\ \mathrm{Inhibition}=\frac{Absorbance\ of\ Control- Absorbance\ of\ Test}{Absorbance\ of\ Control}\ast 100\% $$

### Lipase inhibition activity

The inhibitory potential against lipase was determined by using p-NPB as a substrate [35]. The lipase enzyme was prepared in 0.1 M phosphate buffer saline, pH 8.0 and p-NPB was prepared in 0.1 mM in ethanol. In brief, different concentrations of plant extracts were mixed with 50 μg/mL of the lipase enzyme and incubated for 15 min at 37 °C. 10 μL of p-NPB was added and the reaction volume was maintained to 2 mL with the addition of phosphate buffer and proceeded for 15 min of additional incubation at 37 °C. The absorbance was measured at λ = 405 nm in a UV-visible spectrophotometer. 1% DMSO and Orlistat were used as positive control and reference respectively. The percentage inhibition of lipase was calculated as follows.

$$ \%\mathrm{Lipase}\ \mathrm{Inhibition}=\frac{Absorbance\ of\ Control- Absorbance\ of\ Test}{Absorbance\ of\ Control}\ast 100\% $$

### Tyrosinase inhibition activity

L-DOPA substrate was used to determine tyrosinase ihibition activity, accordingly with the procedure followed by Petrillo et al. [36] with slide modifications. In brief, plant extract of different concentrations in potassium phosphate buffer (0.05 M, pH 6.5) were mixed with 20 μL of mushroom tyrosinase (1000 U/mL) and incubated at 27 °C for 10 min. Then, 200 μL of L-DOPA (5 mM) was added to the mixture and the reaction value was maintained to 2 mL by the addition of phosphate buffer and incubated further for 30 min. Absorbance was measured at λ = 492 nm in a UV-visible spectrophotometer. The phosphate buffer and Kojic acid were used as positive control and reference respectively. The percentage of tyrosinase inhibition was calculated as follows.

$$ \%\mathrm{Tyrosinase}\ \mathrm{Inhibition}=\frac{Absorbance\ of\ Control- Absorbance\ of\ Test}{Absorbance\ of\ Control}\ast 100\% $$

### Elastase inhibition activity

AAAPVN substrate was used to determine elastase inhibition activity, accordingly with the procedure of Liyanaarachchi et al. [33] with slight modification. In brief, the plant extracts of different concentrations were incubated with PPE (0.05 U/mL) for 20 min and 50 μg/mL AAAPVN was added to the reaction mixture and proceeded for 30 min of additional incubation at 25 °C. The final reaction volume was maintained to 200 μL with the addition of 0.2 M Tris-HCL buffer (pH 8.0). The absorbance was taken in λ = 410 nm in a 96-microplate reader (BioTek, EPOCH). 1% DMSO and Quercetin were used as positive control and reference. The percentage of elastase inhibition was calculated as follows.

$$ \%\mathrm{Elastase}\ \mathrm{Inhibition}=\frac{Absorbance\ of\ Control- Absorbance\ of\ Test}{Absorbance\ of\ Control}\ast 100\% $$

### Cholinesterase inhibition activity

Acetylcholinesterase (AChE) and butyrylcholinesterase (BChE) inhibition assays were performed accordingly with Samaradivakara et al. [37] with some modifications. At first, 0.05 U/mL of AChE or 0.5 U/mL of BChE, and plant extract of different concentrations were mixed and incubated at 25 °C for 15 min. Then, 1 mM acetylthiocholine iodide or 1.5 mM of butyrylcholine iodide and 0.5 mM DNTB were added to the reaction mixture followed by an additional 10 min of incubation. The total reaction volume was maintained to 200 μL by the addition of 0.1 M sodium phosphate buffer (pH 8.0). The absorbance was measured at λ = 412 nm in a 96-microplate reader. 1% DMSO and Galantamine were used as positive control and reference respectively. The percentage of cholinesterases inhibition was calculated as follows.

$$ \%\mathrm{AChE}\ \mathrm{or}\ \mathrm{BChE}\ \mathrm{Inhibition}=\frac{Absorbance\ of\ Control- Absorbance\ of\ Test}{Absorbance\ of\ Control}\ast 100\% $$

### High resolution mass spectrometric (HRMS) profiling of metabolites

Liquid chromatography and mass spectrometry (LC-MS) analysis were performed using an HPLC-ESI-QTOF-MS instrument on an Agilent 6520 quadrupole time-of-flight (QTOF) mass spectrometer connected with Agilent 1200-HPLC system via Dual electrospray ionization (ESI) interface (Agilent Technologies, USA). 1 mg/mL stock solution of methanol extract was filtered through a 0.22 μm syringe filter. The filtered stock solution was further diluted to 500 ppm using methanol. The prepared dilution was transferred into a high-performance liquid chromatography (HPLC) autosampler vial for LC-MS analysis and 1 μL aliquot was injected into the HPLC-ESI-QTOF-MS system. The Mass Hunter software version B.04.00 build 4.0.479.0 (Agilent Technology) was used to analyze chromatogram, mass spectra, exact mass calculation, and prediction of chemical formula of the identified compounds as by Singh et al. [38].

### Statistical analysis

All the experiments were carried out triplicates and data were presented in mean ± standard deviation (mean ± SD). Inhibitory concentration at which absorbance is 50% (IC_50_) values were calculated in MS Excel 2013 by linear regression analysis of percentage inhibitions.

## Results

### Antioxidant activities

In our study, crude extracts of *B. pacumbis* showed considerable antioxidant activities against DPPH and ABTS in all solvent extracts with respect to standard Gallic acid (Table 1). The highest antioxidant was found in ethyl acetate extract against DPPH (IC_50_ = 30.14 ± 0.41 μg/mL) and ABTS (IC_50_ = 17.38 ± 1.12 μg/mL) and least was found in hexane extract against DPPH (IC_50_ = 194.41 ± 0.62 μg/mL) and ABTS (IC_50_ = 100.57 ± 0.40 μg/mL) whereas crude methanol and water extract showed satisfactory inhibitory potential against both DPPH and ABTS.

**Table 1:** Antioxidant Activities of *B. pacumbis*

Extracting Solvents	IC_**50**_ Value (μg/mL)	
	DPPH	ABTS
**Hexane**	194.41 ± 0.62	100.57 ± 0.40
**Ethyl Acetate**	30.14 ± 0.41	17.38 ± 1.12
**Methanol**	40.87 ± 0.32	19.03 ± 2.51
**Water**	98.29 ± 0.13	44.28 ± 0.38
**Gallic Acid**	5.12 ± 0.12	1.96 ± 0.05

*IC*_*50*_ Inhibitory concentration at which absorbance is 50%

### Alpha-amylase, alpha-glucosidase, and lipase inhibition activity

**Fig. 1:**
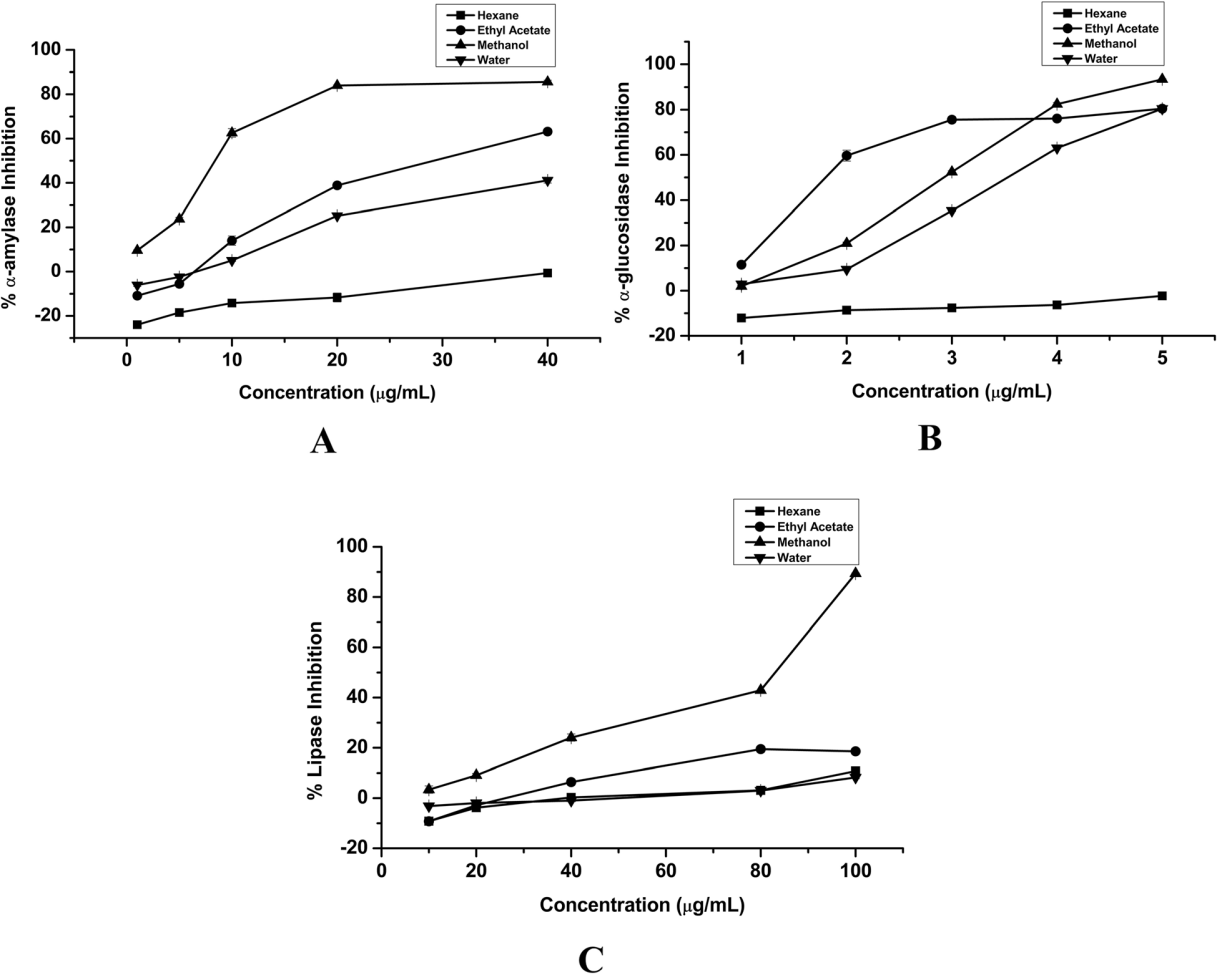
**a***α-*amylase Inhibitory Concentration, **b***α-*glucosidase Inhibitory Concentration, **c** Lipase Inhibitory Concentration of *B. pacumbis*

Our results revealed that crude extracts of *B. pacumbis* in different extracting solvents are potent *α-*amylase and *α-*glucosidase inhibitors (Fig. 1 A&B). The highest *α-*amylase inhibition was found in methanol extract (IC_50_ = 14.03 ± 0.04 μg/mL) and least in water extract (IC_50_ = 43.77 ± 0.54 μg/mL). Similarly, highest *α-*glucosidase inhibition was found in methanol extract (IC_50_ = 0.29 ± 0.00 μg/mL) and least in water extract (IC_50_ = 3.54 ± 0.00 μg/mL) and there is no sign of *α-*amylase and *α-*glucosidase inhibitory potential of hexane extract (Table 2). These results indicate that inhibition of *α-*amylase and *α-*glucosidase by the crude methanol extract of *B. pacumbis* is comparable to the standard drug Acarbose. Furthermore, all solvent extracts of *B. pacumbis* exhibited the lipase inhibitory potential (Fig. 1 C, Table 2). The highest lipase inhibition was found in crude methanol extract (IC_50_ = 90.53 ± 0.31 μg/mL) and least in hexane extract (IC_50_ = 447.86 ± 19.58 μg/mL) which is compared with the standard drug, Orlistat that have an IC_50_ value of 431.58 ± 13.82 μg/mL.

**Table 2:** Enzymes Inhibition Activities of *B. pacumbis*

Extracting Solvents	IC_**50**_ Values (μg/mL)						
	***α-***Amylase	***α-***Glucosidase	Lipase	Tyrosinase	Elastase	AChE	BChE
**Hexane**	NI	NI	447.86 ± 19.58	NI	NI	NI	NI
**Ethyl Acetate**	29.91 ± 0.22	2.49 ± 0.01	121.71 ± 5.75	280.36 ± 1.56	174.34 ± 1.44	36.79 ± 2.66	14.83 ± 1.88
**Methanol**	14.03 ± 0.04	0.29 ± 0.00	67.26 ± 0.17	58.25 ± 1.63	74.00 ± 3.03	31.52 ± 0.58	11.69 ± 0.14
**Water**	43.77 ± 0.54	3.54 ± 0.00	445.202 ± 2.15	168.81 ± 0.56	NI	104.37 ± 1.88	76.99 ± 0.37
**Acarbose**	20.12 ± 0.12	261.23 ± 9.10	–	–	–	–	–
**Kojic Acid**	–	–	–	18.39 ± 0.15	–	–	–
**Orlistat**	–	–	431.58 ± 13.82	–	–	–	–
**Quercetin**	–	–	–	–	101.23 ± 0.16	–	–
**Galantamine**	–	–	–	–	–	1.09 ± 0.02	26.27 ± 1.41

*AChE* Acetylcholinesterase, *BChE* Butyrylcholinesterase, *NI* No inhibition, *IC*_*50*_ Inhibitory concentration at which absorbance is 50%

### Tyrosinase and elastase inhibition activity

**Fig. 2:**
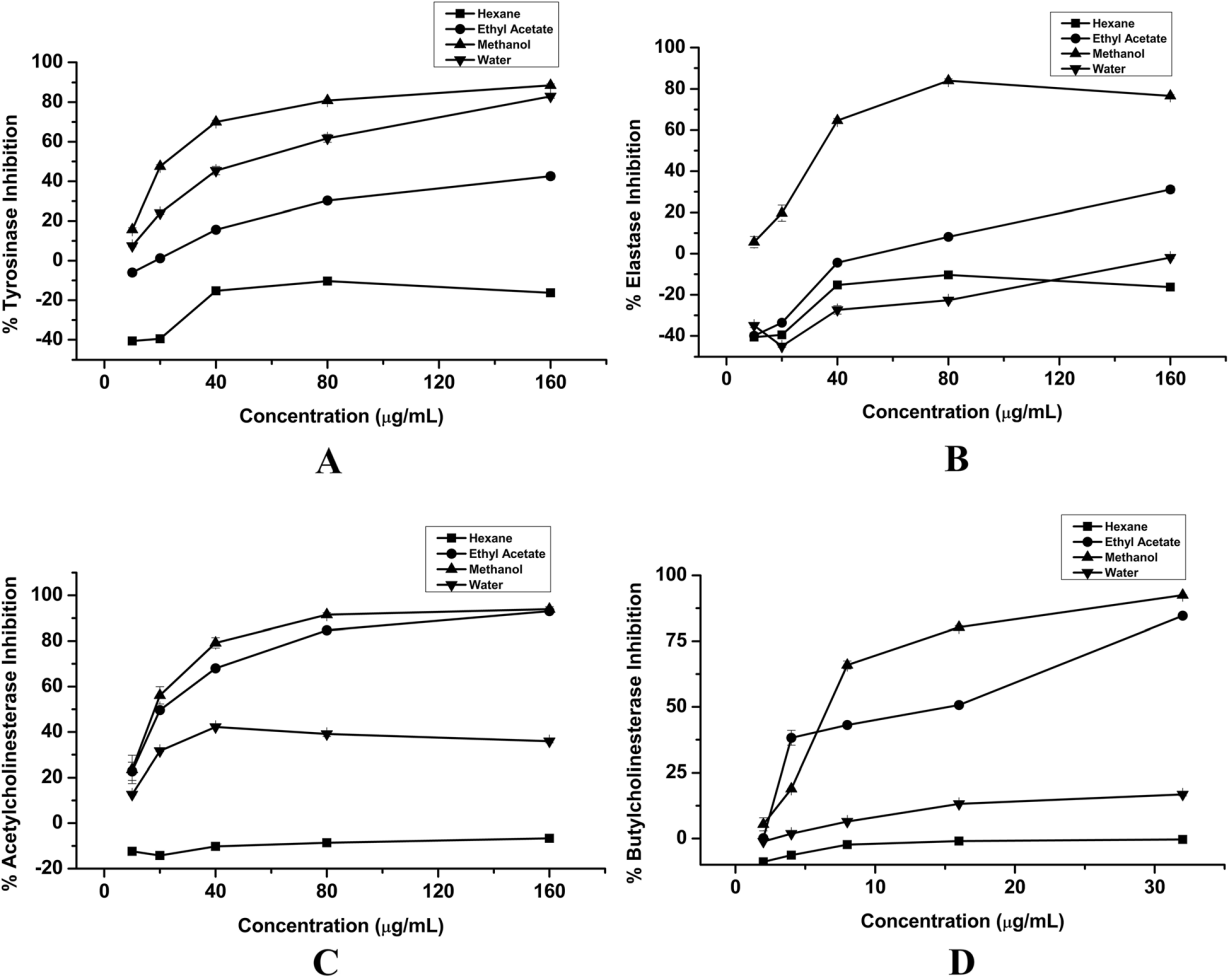
**a** Tyrosinase Inhibitory Concentration, **b** Elastase Inhibitory Concentration, **c** Acetylcholinesterase Inhibitory Concentration, and **d** Butyrylcholinesterase Inhibitory Concentration of *B. pacumbis*

Inhibitions of the tyrosinase enzyme somehow lower the excessive production of melanin and then prevent melanogenesis. Therefore, the control of melanogenesis is important to individuals with clinical or cosmetic needs to enhance dermatological protection. Plant-based natural antioxidants are now gaining much emphasis on modern cosmetics to encounter these skin-related complications. *B. pacumbis* showed higher inhibitory potential against the mushroom tyrosinase in crude methanol extract (IC_50_ = 58.25 ± 1.63 μg/mL) and crude water extract (IC_50_ = 168.81 ± 0.56 μg/mL) with compared to crude ethyl acetate and hexane extract (Fig. 2 A). Kojic acid has greater inhibiting potential as compared to the plant extracts with an IC_50_ value of 18.39 ± 0.15 μg/mL (Table 2). In addition, our results revealed the highest elastase inhibition in crude methanol extract followed ethyl acetate extract as an IC_50_ value of 74.00 ± 3.03 μg/mL and 174.34 ± 1.44 μg/mL respectively (Table 2). Nevertheless, hexane and water extracts of *B. pacumbis* did not show any inhibition against elastase (Fig. 2 B).

### Acetylcholinesterase and butyrylcholinesterase inhibition activity

The examined plant extracts showed inhibitory activities against acetylcholinesterase (AChE) as well as butyrylcholinesterase (BChE). The highest AChE and BChE inhibition were observed in crude methanol extract of *B. pacumbis* as an IC_50_ value of 31.52 ± 0.58 μg/mL and 11.69 ± 0.14 μg/mL respectively followed by crude ethyl acetate and water extract (Table 2). The crude ethyl acetate and water extract also possessed the greater inhibitory potential of these enzymes compared with standard drug, Galantamine (Fig. 2 C&D).

### High resolution mass spectrometry analysis

**Fig. 3:**
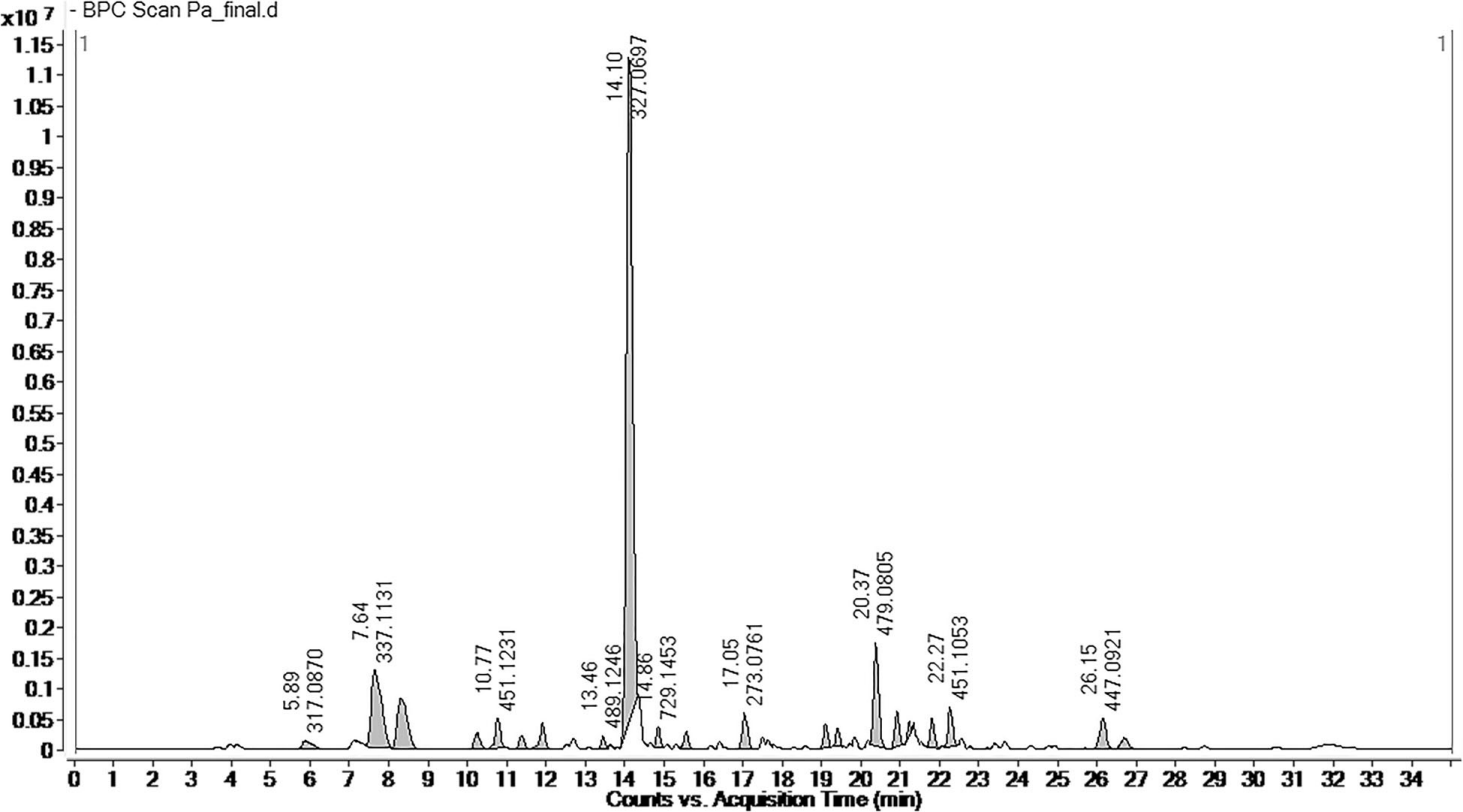
HRMS Chromatogram for Methanol Extract of *B. pacumbis*

**Fig. 4:**
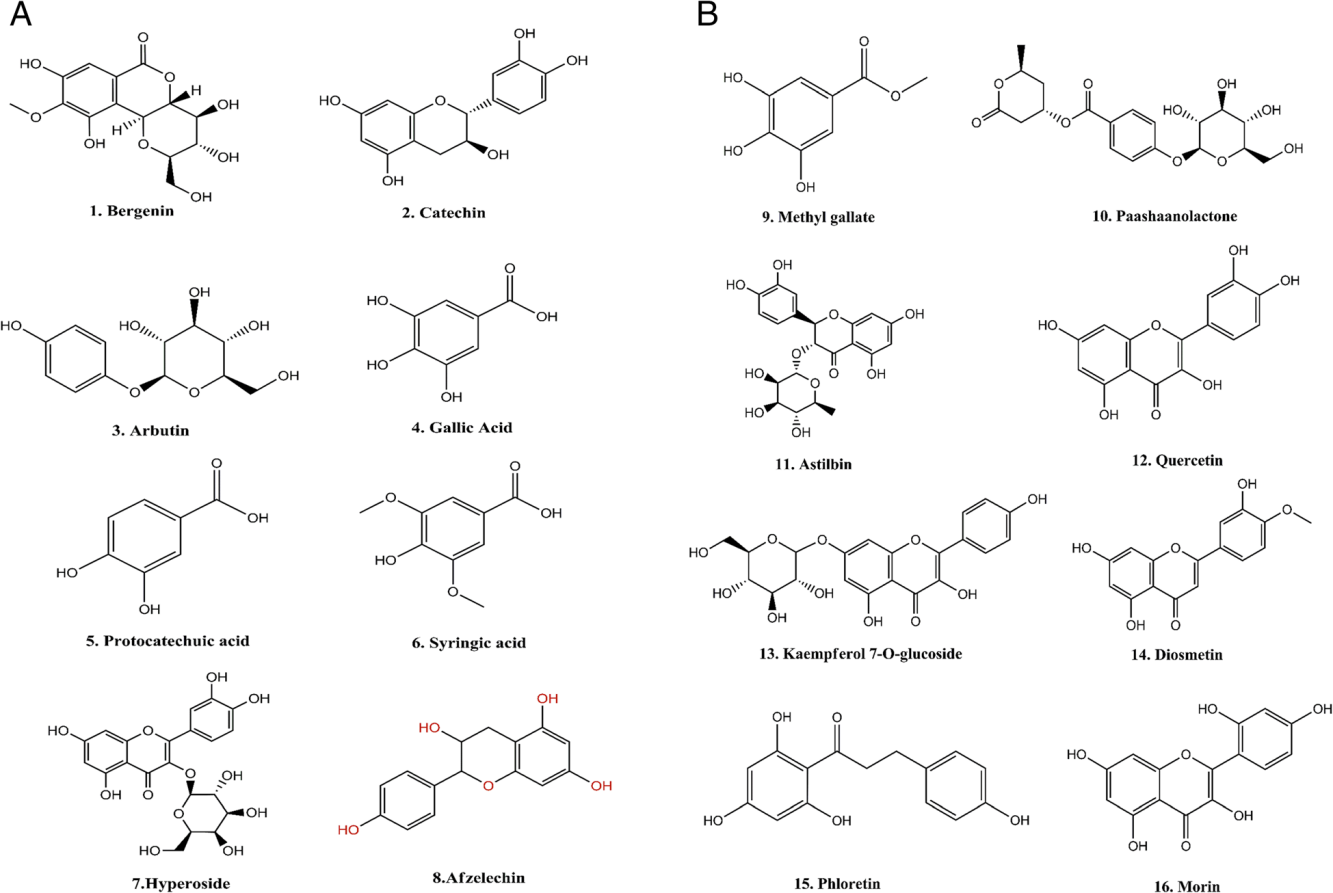
**a** & **b**) Identified Metabolites from *B. pacumbis*

The chromatogram of methanolic extract of *B. pacumbis* showed the different mass spectra of the compounds and we analyzed it further for the prediction of chemical formula including the exact mass calculation by Mass Hunter software version B.04.00 build 4.0.479.0 (Agilent Technology) (Fig. 3). Detail of identified compounds with their theoretical and observed mass to charge ratio and errors in parts per million (ppm) in positive ion mode in ESI is presented in Table 3s. The compounds were identified based on the observed MS spectra and also compared with the literature data [39–41]. We observed the presence of flavonoids and phenolic compounds in the crude methanol extract of *B. pacumbis* such as Bergenin (m/z = 327.072), Catechin (m/z = 289.071), Arbutin (m/z = 271.082), Gallic acid (m/z = 169.014), Protocatechuic acid (m/z = 153.019), Syringic acid (m/z = 197.045), Hyperoside (m/z = 463.088), Afzelechin (m/z = 273.076), Methyl gallate (m/z = 183.029), Paashaanolactone (m/z = 411.129), Astilbin (m/z = 449.108), Quercetin (m/z = 301.034), Kaempferol-7-O-glucoside (m/z = 447.093), Diosmetin (m/z = 299.056), Phloretin (m/z = 273.076), and Morin (m/z = 301.035) (Fig. 4 A&B).

**Table 3:** Details on Metabolites Identified by HRMS

S. No.	Compounds	Molecular Formula	Molecular weight [M]	Measured Mass (M-H)^**−**^	Observed Mass (M-H)^**−**^	Retention Time (minutes)	Error (ppm)
1	Bergenin	C_14_H_16_O_9_	328.273	327.072	327.072	14.00	1.22
2	Catechin	C_15_H_14_O_6_	290.260	289.071	289.071	17.60	0.62
3	Arbutin	C_12_H_16_O_7_	272.253	271.082	271.082	5.80	0.06
4	Gallic acid	C_7_H_6_O_5_	170.120	169.014	169.014	8.30	0.57
5	Protocatechuic acid	C_7_H_6_O_4_	154.121	153.019	153.019	12.50	1.25
6	Syringic acid	C_9_H_10_O_5_	198.174	197.045	197.045	12.70	1.02
7	Hyperoside	C_21_H_20_O_12_	464.379	463.088	463.087	24.70	0.72
8	Afzelechin	C_15_H_14_O_5_	274.272	273.076	273.076	17.10	1.93
9	Methyl gallate	C_8_H_8_O_5_	184.150	183.029	183.029	15.70	1.91
10	Paashaanolactone	C_19_H_24_O_10_	412.136	411.129	411.129	21.30	0.39
11	Astilbin	C_21_H_22_O_11_	450.396	449.108	449.108	17.80	1.68
12	Quercetin	C_15_H_10_O_7_	302.236	301.035	301.034	25.70	1.82
13	Kaempferol-7-O-glucoside	C_21_H_20_O_11_	448.380	447.093	447.093	26.00	0.33
14	Diosmetin	C_16_H_12_O_6_	300.266	299.056	299.055	18.70	1.82
15	Phloretin	C_15_H_14_O_5_	274.260	273.076	273.076	19.80	0.51
16	Morin	C_15_H_10_O_7_	302.235	301.035	301.035	25.60	0.34

## Discussion

Plants has been used for medicine from ancient times for the treatment of different human ailments. Antioxidant compounds act as a preventive agent against oxidation process. Antioxidants scavenge free radicals and protect humans against various diseases. Nowadays lots of synthetic molecules with potential enzyme inhibitory tendencies are being used as a food supplement. Although synthetic antioxidants are in use, it might have adverse health effects due to their several side effects [42]. Hence, more researches are being carried out to identify the potential natural antioxidant molecules from herbal extract [43]. In this study, *B. pacumbis* root was extracted with four different solvent hexane, ethyl acetate, methanol, and water. Antioxidant capacities of the solvent extract were analyzed using DPPH and ABTS assay. Results revealed that crude ethyl acetate and methanol extract have the highest antioxidant capacities with the least IC_50_ values as depicted in results. Furthermore, crude hexane extract showed the lowest antioxidant tendencies as compared to the crude water extract. The results are comparable with reported *Bergenia* species [44, 45]. Based on this finding we suggested that different crude extracts of roots of *B. pacumbis* are the considerable source of antioxidant molecules and can be extracted and analyzed further in-vivo for the several health benefits. The different phenolics and flavonoids molecules present in the plant extract might be the source of antioxidant activities [6]. We next examined the plant extracts for their inhibitory potential towards *α-*amylase, *α-*glucosidase, lipase, tyrosinase, elastase, acetylcholinesterase, and butyrylcholinesterase.

Obesity is linked with the abnormal accumulation of fat in the body and causes several health complications such as cardiovascular diseases, cancers, osteoarthritis, hypertension and diabetes [46]. Scientific evidence supported that the person suffering from obesity and diabetes are more prone to the development of cardiovascular disease [47]. It has been proven that medicinal plant and its extract has tendencies to lower the risk of obesity and diabetes by inhibiting the enzymes such as lipase, *α-*amylase, and *α-*glucosidase that are involved in fat and carbohydrates metabolism in the human body. The *α-*amylase and *α-*glucosidase are the carbohydrate hydrolyzing enzyme that breakdown the glycosidic bond and releases glucose, which increases the concentration of glucose in the body and has a negative impact on diabetic patients. The pathogenesis of T2DM is currently accredited to genetics and metabolic abnormalities [48] and its treatment with the currently available drugs is still not adequate to prevent long-term diabetic complications [49]. Moreover, human pancreatic lipase is the main enzyme that breaks down dietary fats in the human digestive system [20, 50]. The commercial anti-obesity drugs have some serious side effects [51, 52] which enforce plant-based remedies of natural choice for the treatment of obesity that considered to have wide variety of natural chemical compounds having diverse structural features to inhibit lipase [53]. Plant extract had tendencies to inhibit the digestive enzyme such as *α-*amylase, *α-*glucosidase, and lipase which made medicinal plant and herbal remedies as alternative source of inhibitor of major enzymes and gaining public interest across the world. Different species of *Bergenia* and their secondary metabolites are already reported as potent anti-diabetic sources in-vitro and in-vivo [54–57]. Our results revealed that crude extracts of *B. pacumbis* are the inhibitor of these digestive enzymes. The root extract of *B. pacumbis* showed inhibitory activities towards *α-*amylase, *α-*glucosidase, and lipase. Results revealed that among four analyzed solvent extracts, the crude methanol extract has greater inhibitory activities compared to Acarbose and Orlistat. These results are consistent with the investigation of the different fractions of *B. ciliata* by Bhandari et al., 2007 [54]. However, hexane extract does not reveal any inhibition against these enzymes. Our finding opens up the possibility of finding potential inhibitors in the methanol extract.

Melanin is an important component which helps in regulating physiology, pathology, and toxicology of several organs such as skin, eyes, and brain [58]. Accumulation of unnecessary levels of epidermal pigmentation causes various dermatological disorders, such as age spots and freckles [59]. Due to the high reactivity of tyrosinase, melanin reacts with amino acids and proteins to enhance the brown color pigmentation in the skin [60, 61]. Scavenging of ROS and tyrosinase enzyme by natural bioactive molecules might be an option to enhance the whitening of skin color [62]. The generation of ROS by solar ultraviolet (UV) radiation adversely affects skin health by the activation of enzymes such as elastase that degrade extracellular matrix (ECM) proteins in the dermis [63]. Thus, elastase inhibition is a useful approach to prevent skin alterations and premature skin aging. In our observation, crude methanol extract followed by water and ethyl acetate extract of *B. pacumbis* has tyrosinase inhibitory potential compared to standard drug Kojic acid. In comparison to the lipase, amylase and glucosidase inhibitory potential, crude extracts of *B. pacumbis* revealed comparatively lower inhibitory potential against tyrosinase and elastase. Only crude methanol and ethyl acetate extracts of *B. pacumbis* showed the elastase inhibition compared to standard Quercetin. Whereas, crude water extract does not reveal any inhibition in the given concentration towards elastase enzymes. The presence of arbutin might be the defined source of tyrosinase inhibition from *B. pacumbis* [64, 65]. However, the purification of those extracts might enhance the inhibitory potential of *B. pacumbis* on the tyrosinase and elastase.

In recent years, enzyme inhibitory strategies are considered as one of the most effective strategies in combating global health problems including Alzheimer’s diseases (AD) [66]. The natural inhibitors of cholinesterases (ChEs) from the plant origin for the management of cognitive/mental disorders have gained interest due to the presence of polyphenolic compounds such as quercetin, catechin, bergenin, and rutin which have several health benefits [67, 68]. Aromatic and medicinal plants may have an important role in oxidative stress protection, which are good sources of acetylcholinesterase and butyrylcholinesterase inhibitor to controlling AD [69]. A recent study showed that the different pure isolated metabolites from *B. ciliata* have potent acetylcholinesterase and butyrylcholinesterase inhibition activity [44]. Our study revealed that crude methanol, ethyl acetate, and water extract of *B. pacumbis* to have great inhibitory potential toward acetylcholinesterase and butyrylcholinesterase. However, hexane extract does not reveal any inhibition towards these enzymes. Remarkably methanol extract has significant inhibitory activities against all enzymes.

The diverse secondary metabolites with ranges of pharmacological significance were isolated and studied from *Bergenia* species. For example gallic acid, tannic acid, glucose, mucilage, bergenin, stigmasterol, β-sitosterol, arbutin, phytol, damascenone, 3-methyl-2-buten-1-ol, syringic acid, hyperoside, afzelechin, methyl gallate, paashaanolactone, etc. have been isolated from *Bergenia ciliata*, *Bergenia ligulatas*, and *Bergenia stracheyi* till date [7, 70, 71]. Our HRMS data also revealed the presence of diverse secondary metabolites in the methanol extract, which might be the reason behind the higher bioactivities. These secondary metabolites possess marked bioactivities. Enzymes inhibitory tendencies of these extracts may be due to the presence of diverse bioactive molecules such as catechin, bergenin, and many other flavonoids molecules. Bergenin and catechin are the marker compound of *Bergenia* species which are major bioactive ingredients in the herb–drug [39, 72] and have antioxidant, anti-inflammatory, antiviral, antihyperglycemic, immunostimulant, and antipyretic potential [39, 73]. Phenolic acids such as gallic acid, protocatechuic acid, and ferulic acid are known to inhibit cancer cells [74]. The major compound identified in *B. pacumbis* were Bergenin, catechin, arbutin, gallic acid, protocatechuic acid, syringic acid, hyperoside, afzelechin, methyl gallate, paashaanolactone, astilbin, quercetin, kaemferol-7-O-glucoside, diosmetin, phloretin, and morin. Most of the compounds identified here are highly abundant in *Bergenia* species. Arbutin is the great tyrosinase inhibitor [65], morin has great antioxidant and cholinesterases inhibiting potential [44]. Flavonoids like afzelechin and quercetin show antioxidant and anti-diabetic properties [75, 76]. All the identified compounds based on HRMS data were further verified with the literature report i.e. Arbutin, Gallic Acid, Protocatechuic Acid, Bergenin, Catechin and Syringic Acid [39], Hyperoside [77], Afzelechin [78, 79], Methyl Gallate [80], Paashaanolactone [81], Astilbin [82], Quercetin [83], Kaempferol-7-O-glucoside [84, 85], Diosmetin [86], Phloretin [87], and Morin [88]. We firmly believe that presence of phenolic and flavonoids molecules are largely responsible for the bioactivities of *B. pacumbis*.

## Conclusion

Our study provides a plethora of scientific evidence that the different extracts of *B. pacumbis* from Nepali origin have astonishing potential on inhibiting free radicals as well as enzymes involved in digestion, skin related problems, and neurological disorders compared with the commercially available drugs. The great ability of this plant to inhibit those enzymes is basically due to the presence of active secondary metabolites. Our finding opens up the possibilities in future to identify the potent inhibitory compounds of pharmaceuticals and cosmetics application.

## Data Availability

The datasets used and/ or analyzed during the current study available from the corresponding author on reasonable request.

## Abbreviations

μg: Microgram
AAAPVN: N-Succinyl-Ala-Ala-p-nitroanilide
ABTS: 2, 2′-Azino-bis (3-ethylbenzothiazoline-6-sulfonic acid) diammonium salt
AChE: Acetylcholinesterase
AD: Alzheimer’s disease
BChE: Butyrylcholinesterase
DM: Diabetes mellitus
DMSO: Dimethyl sulfoxide
DNSA: 3,5-Dinitrosalicylic acid
DPPH: 2, 2-Diphenyl-1-picrylhydrazyl
DTNB: 5,5′-dithiobis-(2-nitrobenzoic acid)
ECM: Extracellular matrix
ESI: Electrospray ionization
HPLC: High-performance liquid chromatography
HRMS: High resolution mass spectroscopy
IC_50_: Inhibitory concentration at which absorbance is 50%
KATH: National Herbarium and Plant Laboratory
KOICA: Korea International Cooperation Agency
LCMS: Liquid chromatography and mass spectrometry
L-DOPA: l-3,4-dihydroxyphenylalanine
M: Molar
mL: Milliliter
mM: Millimolar
NAST: Nepal Academy of Science and Technology
p-NPB: 4-nitrophenyl butyrate
p-NPG: p-Nitrophenyl-α-D-glucopyranoside
PPE: Porcine pancreatic elastase
ppm: Parts per million
QTOF: Quadrupole time-of-flight
ROS: Reactive oxygen species
RSA: Radical scavenging activity
U: Units
UV: Ultraviolet
